# Ventricular Arrhythmias in Ischemic Cardiomyopathy—New Avenues for Mechanism-Guided Treatment

**DOI:** 10.3390/cells10102629

**Published:** 2021-10-01

**Authors:** Matthew Amoni, Eef Dries, Sebastian Ingelaere, Dylan Vermoortele, H. Llewelyn Roderick, Piet Claus, Rik Willems, Karin R. Sipido

**Affiliations:** 1 Experimental Cardiology, Department of Cardiovascular Sciences, KU Leuven, 3000 Leuven, Belgium; matthew.amoni@kuleuven.be (M.A.); eef.dries@kuleuven.be (E.D.); sebastian.ingelaere@kuleuven.be (S.I.); llewelyn.roderick@kuleuven.be (H.L.R.); Rik.Willems@kuleuven.be (R.W.); 2 Division of Cardiology, University Hospitals Leuven, 3000 Leuven, Belgium; 3 Department of Medicine, Faculty of Health Sciences, University of Cape Town, Cape Town 7935, South Africa; 4 Imaging and Cardiovascular Dynamics, Department of Cardiovascular Sciences, KU Leuven, 3000 Leuven, Belgium; dylan.vermoortele@kuleuven.be (D.V.); piet.claus@kuleuven.be (P.C.)

**Keywords:** arrhythmias, myocardial infarction, hypertrophy, fibrosis, cardiac remodelling, calcium, action potential

## Abstract

Ischemic heart disease is the most common cause of lethal ventricular arrhythmias and sudden cardiac death (SCD). In patients who are at high risk after myocardial infarction, implantable cardioverter defibrillators are the most effective treatment to reduce incidence of SCD and ablation therapy can be effective for ventricular arrhythmias with identifiable culprit lesions. Yet, these approaches are not always successful and come with a considerable cost, while pharmacological management is often poor and ineffective, and occasionally proarrhythmic. Advances in mechanistic insights of arrhythmias and technological innovation have led to improved interventional approaches that are being evaluated clinically, yet pharmacological advancement has remained behind. We review the mechanistic basis for current management and provide a perspective for gaining new insights that centre on the complex tissue architecture of the arrhythmogenic infarct and border zone with surviving cardiac myocytes as the source of triggers and central players in re-entry circuits. Identification of the arrhythmia critical sites and characterisation of the molecular signature unique to these sites can open avenues for targeted therapy and reduce off-target effects that have hampered systemic pharmacotherapy. Such advances are in line with precision medicine and a patient-tailored therapy.

## 1. The Health Challenge of Arrhythmias in Ischemic Heart Disease

Ischemic heart disease (IHD) is the leading cardiovascular disease and largest single cause of death in the US and Europe, accounting for up to ~20% of deaths [1,2]. A significant proportion of this mortality is due to sudden cardiac death (SCD), where combining acute and chronic mortality it can be estimated that approximately 50% of deaths in IHD are due to SCD, with the other half due to heart failure and other complications [3,4]. SCD is attributed to lethal ventricular arrhythmias of ventricular tachycardia (VT) and fibrillation (VF), which are common complications of IHD in the acute, chronic and heart failure stage [5].

**Figure 1:**
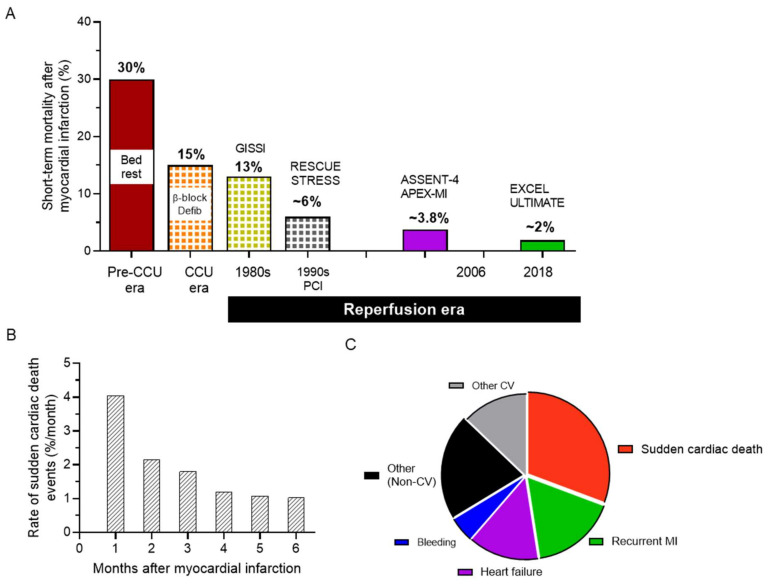
Sudden cardiac death and ischemic heart disease (IHD). (**A**) Estimated short-term mortality following myocardial infarction (Adapted from [7] and respective clinical trials). β-block–β-blocker therapy; Defib–defibrillation; CCU–coronary care unit; PCI–percutaneous coronary intervention. (**B**) Sudden cardiac death accounts for the largest proportion of death in IHD (Adapted from [5]). (**C**) Incidence rates of sudden cardiac death events (sudden cardiac death or resuscitated sudden cardiac death) in the first 6 months after myocardial infarction (Adapted from [3]). CV–cardiovascular.

IHD has evolved dramatically in presentation over the decades. The primary pathology remains coronary artery disease due to atherosclerosis. Narrowing of coronary arteries leads to intermittent ischaemia, and complete coronary artery occlusion occurs when thrombosis superimposes on a vulnerable lesion leading to myocardial infarction (MI) [6]. Before proper treatment, immediate mortality of acute coronary occlusion and MI was over 30% (Figure 1A). This includes a substantial number of patients for whom SCD was the first manifestation of IHD. The immediate management of MI has evolved from the expectant conservative in the 1950s through the medical management revolution of thrombolysis in the 1980s [7], to the current status of immediate revascularisation by primary percutaneous coronary interventions. As illustrated in Figure 1A, under current guidelines, the acute mortality is now well below 5% [8]. This reduction has led to new presentations of IHD. Survivors of MI, with substantial loss of myocardial tissue, develop ischemic cardiomyopathy (ICM) and remain at high risk of premature death because of evolution to heart failure and because of arrhythmia risk. Arrhythmic death was highest in the first 30 days after MI in the VALIANT cohort (Figure 1B), and related to left ventricular dysfunction [5]. A recent meta-analysis of 14 trials conducted in NSTEMI, reported SCD as the major cause of death and high risk of arrhythmias early but even more so after 30 days, indicating the presentation and evolution of the MI has an important role (Figure 1C) [3]. This poses new challenges. Management of arrhythmias in ICM is a major problem as risk assessment and pharmacological management are severely limited.

Development of novel therapeutics for ventricular arrhythmias has been stagnant, partly related to disappointments in late stage clinical trials, possibly also in part due to some complacency with available device therapy [9]. Another hurdle may also be that, despite many preclinical studies, our understanding of basic cellular pathophysiology and arrhythmia mechanisms in the infarcted heart remains incomplete. In this review, we discuss the unique nature of the presentation of arrhythmias in the presence of a healed MI and summarise what we know about electrical remodelling in the chronic phase after MI. The various factors that contribute to arrhythmia vulnerability are discussed in the context of therapeutic targeting. Finally, we present the gaps in knowledge and future research aims.

## 2. Current Management: Much to Be Desired from Pharmacotherapy

Restoring adequate circulation to the ischemic myocardium is cardinal to preserving viability and preventing further loss. Therefore, revascularisation remains central to the immediate and long-term management of IHD [10,11]. It is also important to avoiding adverse remodelling and pathological triggers associated with repetitive, demand ischaemia that can precipitate and facilitate arrhythmias and SCD [12]. Ancillary to revascularisation, cardioprotective therapies have the potential to salvage at-risk myocardium with novel therapies emerging to reduce ischemic and particularly reperfusion injury targeting multifactorial actors in acute MI [13]. Beyond revascularisation, guideline-based therapy includes beta-blockers and inhibition of the RAAS system, to reduce the extent of ventricular remodelling and evolution to heart failure. Early trials for primary pharmacological prevention of arrhythmias had disastrous outcomes with increased mortality in treated patients because of proarrhythmia [14,15]. Although beta-blockers reduce arrhythmias [16,17], they are not sufficient for prevention of SCD. Other anti-arrhythmic drugs available in IHD are amiodarone, possibly ranolazine [9,16]. A recent report suggested the use of agents like quinidine in select patients with refractory short coupled malignant arrhythmias [18], but no new anti-arrhythmics have been developed targeting ventricular arrhythmias in the last two decades that have shown significant promise in making their way into clinical practice.

**Figure 2:**
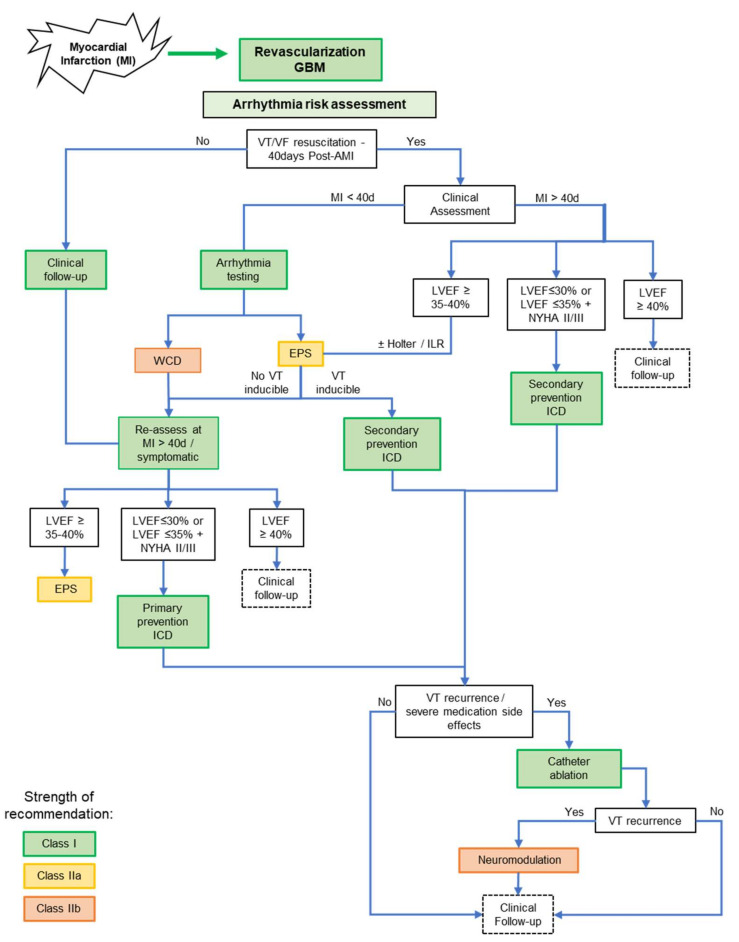
Risk assessment and management of ventricular arrhythmias after myocardial infarction. The flowchart was derived from [16,22,23]. Abbreviations: GBM–Guideline-based medical therapy; VT–ventricular tachycardia; VF–ventricular fibrillation; EPS–Electrophysiological study; WCD–wearable cardioverter-defibrillator; ILR–implantable loop recorder; ICD–implantable cardioverter-defibrillator. LVEF–left ventricular ejection fraction.

For patients who have received the prescribed revascularisation and guideline-based medical therapy, an arrhythmia risk assessment is called for early after MI (2–40 days post-MI) if there is a clinical suspicion of high-risk (syncope, non-sustained VT, etc). Figure 2 illustrates a flowchart based on available guidelines and recommendations. Time after MI, documented VT/VF and left ventricular ejection fraction (LVEF) primarily define the risk and eventual need for an implantable cardioverter defibrillator (ICD) vs. medical optimisation and follow-up. ICD patients with intolerable symptoms or arrhythmia recurrence despite optimal medical therapy require catheter ablation or surgical neuromodulation. Of note, although the early post-MI period is recognised as highest risk (Figure 1), even after years, asymptomatic MI patients can suddenly develop life-threatening arrhythmias, possibly related to evolving disease or comorbidities and require an ICD.

Thus, for patients at high risk, ICD is the primary treatment option and an ICD is considered the most effective therapy for the prevention of SCD in ICM [16,19]. ICDs effectively detect ventricular arrhythmias of sustained VT and VF and deliver a high-energy shock to the heart to restore normal sinus rhythm. Current guidelines for ICD implantation only have consensus regarding risk based on assessment of left ventricular ejection fraction (LVEF) <35% [16]. However, it has long been understood that although the degree of left ventricle (LV) dysfunction is the most powerful predictor of post-MI mortality, this metric is insufficient. This deficiency is further highlighted by the paradoxical evidence that the highest mortality is observed in patients with preserved, or only moderately reduced LVEF [20,21].

Classically, the invasive electrophysiology (EP) study involving arrhythmia provocation by programmed electrical stimulation via intracardiac catheters inserted transvenously is a standard for risk assessment and has its place in the diagnostic workup illustrated in Figure 2 [16,24]. However, its utility in the clinical approach to risk stratification has been challenged, particularly its usefulness as a screening tool [25]. Therefore, ancillary non-invasive tools and parameters have been proposed [26]. Further assessment of ischemia, a potential arrhythmia triggers is recommended by coronary angiography, stress echocardiography and nuclear scans [16,27]. Parameters such as infarct size, and peri-infarct heterogeneity of fibrosis have been reported to predict arrhythmias, but no concrete method of assessment and evidence has found place in clinical practice [28,29]. The 24-h Holter remains one of the clinicians most valuable tool to assess an ICM patient’s risk for SCD, with frequent premature ventricular complexes (PVCs), non-sustained VT and sustained VT or VF providing compelling indicators of high risk and potential benefit of ICD implantation [16,30,31]. Data obtained from ICD support the use of new markers of arrhythmic risk derived from repolarisation abnormalities [32]. A potential Holter-based parameter is beat-to-beat variability of repolarisation that shows promise of translation to ICD monitoring [33,34,35]. Repolarisation parameters derived from standard 12-lead electrocardiogram (ECG) analysis include QT-interval and its dispersion, T-peak to T-end and microvolt T-wave alternans, which may reflect the dynamic substrate for arrhythmias as well as depolarisation, and parameters such as QRS duration and QRS fragmentation that reflect conduction abnormalities [36,37]. These new parameters still need to be assessed comprehensively before recommendation to clinical practice [38].

A main limitation of the ICD, is that although it is effective at terminating arrhythmia, it does not prevent the occurrence of arrhythmia and may contribute to progression of disease. A high incidence of shocks to restore sinus rhythm is associated with worse outcome among ICD patients; this is possibly due to induction of electromechanical dissociation and SCD, and to repeated shocks contributing to progression of adverse remodelling [39,40]. In addition, shocks cause significant psychological distress and negatively impact patient’s quality of life [41]. Thus, strategies to prevent the occurrence of arrhythmia are often used in conjunction to ICD implantation. Electrophysiology study with mapping and catheter ablation of VT is the most effective approach to prevent the occurrence of arrhythmia [42,43]. After identification of areas critical to the arrhythmia maintenance or initiation, ablation using radiofrequency energy interrupts the arrhythmia path or triggering tissue, thereby preventing arrhythmia occurrence [44]. However, ablation success is significantly lower in patients with intramural or epicardial scars and arrhythmia circuits, patients with hemodynamically unstable arrhythmias that preclude mapping or complex scars with multiple exits or polymorphic arrhythmias [45]. Patients with incessant arrhythmias or arrhythmia storm, which is refractory to ablation and medical therapy or where these are contraindicated, are considered for neuromodulation therapy [16,46]. In these patients, bilateral cardiac sympathetic denervation via thoracoscopic minimally invasive therapy has been shown effective with up to 60% success after three years [47], but this complex procedure is yet to be established in routine clinical practice.

Whereas ICD is a symptomatic treatment, ablation as treatment is based on the unique remodelling of the LV after MI and the consequent macroscopic mechanisms of VT. Further development of pharmacotherapy or alternative treatment will need to focus on the mechanistic basis of post-MI arrhythmias.

## 3. The Unique Nature and Central Role of the Border Zone

**Figure 3:**
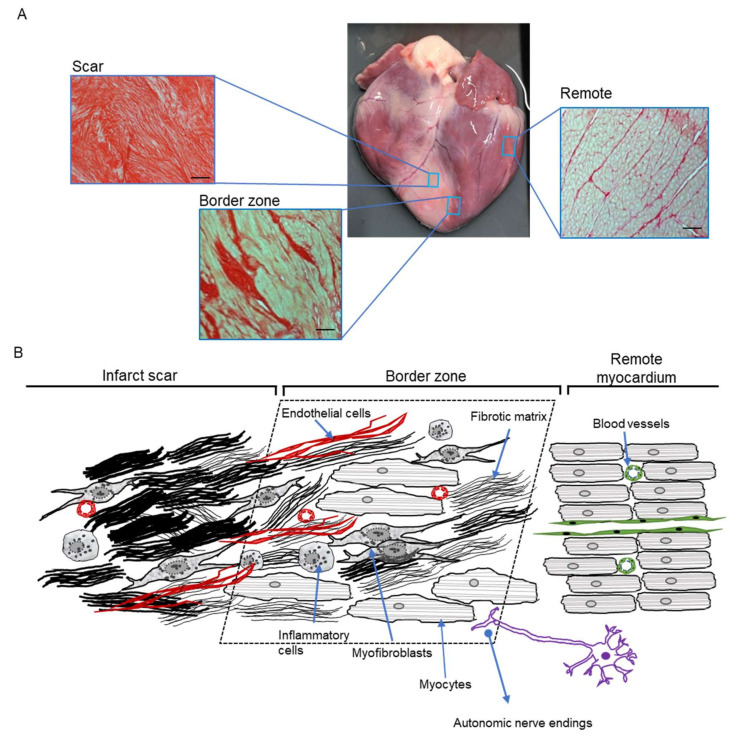
The unique nature of the myocardial infarction border zone. (**A**) Picture of a pig heart from with ischaemia/reperfusion injury-induced myocardial infarction after 1 month, illustrating the histological fibrotic structure of the scar (left insert) the mixed fibrosis and myocytes in the border zone (middle insert) and the healthy/non-infarcted remote myocardium (right insert) by Picosirius red staining (Adapted from [49]). (**B**) Schematic diagram illustrating the multicellular milieu of the border zone as transition between scar and myocardium without ischemic damage (remote myocardium).

After MI, there are three structurally distinct regions in the LV (Figure 3A): the infarct region subjected to ischaemia, and reperfusion in most cases, where necrotic tissue is replaced by fibrosis, with scar formation preserving the wall integrity. Surrounding the infarct is a transition border zone (BZ) which was subject to relative ischaemia and collateral perfusion. Here the fraction of myocytes that survived this insult is intermixed with fibrosis. Third is the remote region that was unaffected by the ischemic insult, but which will eventually have to compensate for the loss of muscle mass.

Our understanding of the pathology and time-course of post-MI repair and remodelling has deepened through experimental studies [48]. The initial phase of MI (day 1–5) is characterised by cell death and inflammation. During this phase rapid myocyte loss occurs by necrosis and apoptosis, with infiltration and activation of immune cells and fibroblasts. The intermediate phase (Day 7–30) is characterised by resolution of the inflammation and replacement of myocyte loss by fibrosis leading to a myocardial infarct or scar. The chronic phase (Day 30 onwards) is the remodelling phase during which the remaining myocytes remodel to adapt to the changes due to the loss of a part of the ventricle.

The BZ formation involves a complex symphony of immune cells, including neutrophils, infiltrating monocytes, regulatory T cells and resident macrophages, as well as pericytes, endothelial cells and fibroblasts interacting directly and indirectly with myocytes in the BZ [48,50,51].

The myocytes in the BZ undergo substantial remodelling in response to different signals. The transient ischaemia leads to activation of survival pathways such as the RISK and SAFE pathways [52,53]. The acute loss of a significant percentage of myocardium in MI results in abrupt and progressive changes of ventricular loading and local wall stress leading to abnormal strain in the surviving border zone and non-infarcted remote region, that contribute to remodelling and altered regional strain [54,55]. The wall stress in the scar and BZ is higher than to that of the remote myocardium primarily due to the difference in thickness [56,57,58], affecting fibroblast properties as well [59]. In addition, the BZ is under unique load during systole, due to the non-compliant scar tissue on one side leading to uneven mechanical forces and contraction [55,56]. This contributes to a BZ-specific myocyte phenotype, different from the remodelling in the remote myocardium [49,55,57,60].

Another important feature of the BZ is the altered nature of the local autonomic innervation. Cardiac sympathetic nerves also suffer ischemic injury and death in the infarct region resulting in denervation [61,62]. Similar processes are observed to a lesser extent in the BZ. Here surviving neurons attempt to regenerate and sprout, leading to a heterogeneous innervation [63,64]. Various nerve growth factors including NGF, GAP43, and protein tyrosine phosphatase sigma (PTP) have been described to regulate neuronal re-innervation and targeting such factors can improve innervation and abate arrhythmias [65,66]. Following MI, the innervation of the heart undergoes remodelling in the form of denervation-re-innervation with aberrant nerve sprouting particularly in the BZ [65,67]. These processes modulate myocyte function and remodelling, influencing ion channel expression and the BZ phenotype [68]. Neurons in the non-infarcted region also undergo neuronal remodelling [69]. Moreover, extra-cardiac neuronal remodelling occurs with observed enlargement and dendritic sprouting of stellate ganglia in MI-models that further influences myocyte remodelling [65,70,71].

Lastly, not only the myocardium and innervating network, but also the perfusing vasculature and microcirculation in the ischemic area are damaged with subsequent remodelling in the reperfused BZ [72,73]. The observations of a rest-stress perfusion defect mismatch in high risk patients confirms that perfusion of the BZ is dysfunctional [74,75]. In the BZ, local vascular damage, stenosis, and angiogenesis occur during infarct healing [76]. These new vessels have abnormalities of smooth muscle and organisation, leading to dysfunction particularly under stress where nitric-oxide and bradykinin-mediated vasodilatation is impaired [77,78,79]. This contributes to the vulnerability to ischaemia of the BZ and consequent arrhythmias.

Taken together, as illustrated in Figure 3B, the BZ microarchitecture, resulting from a complex local remodelling process, forms a unique substrate for arrhythmias in IHD as supported by in vivo studies.

## 4. Origin and Maintenance of VT–Mechanistic Insights Obtained In Vivo

### 4.1. The Conceptual Framework

**Figure 4:**
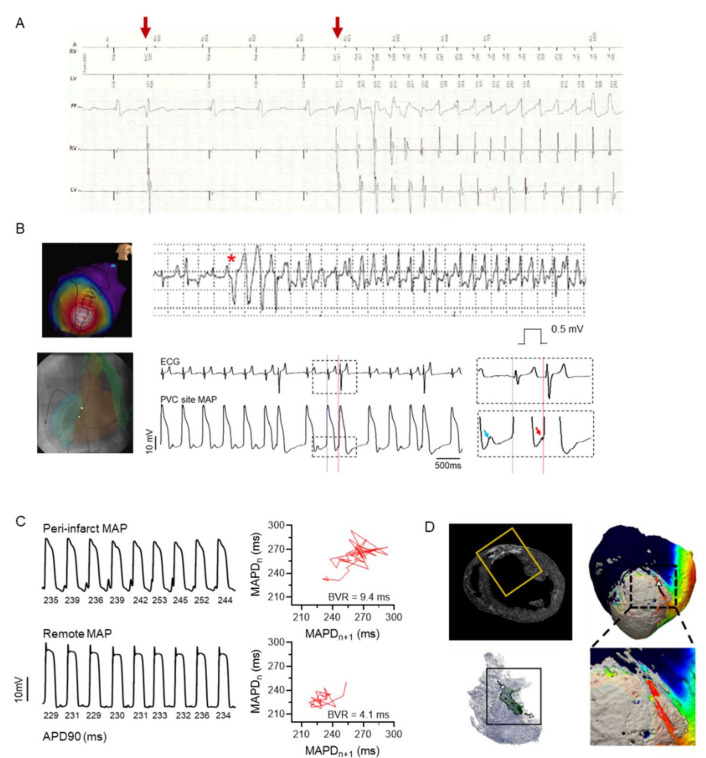
Arrhythmia mechanisms in vivo. (**A**) Example of ventricular arrhythmia initiation by a premature ventricular complex (PVC) from ICD recording of a patient. (**B**) Top: Initiation of ventricular tachycardia by a PVC during increased adrenergic drive from a loop recorder of an awake, freely-moving pig with ICM (top) and mapping of PVCs provoked by adrenergic stimulation in an anesthetised animal. Bottom: probing the site of PVCs utilizing monophasic action potential (MAP) catheters (right) revealed that the dominant mechanism is delayed after depolarisation-triggered activity (Adapted from [49]). (**C**) MAP recording illustrating beat-to-beat variability of repolarisation, a functional substrate, is increased in the border zone during sympathetic stimulation (Adapted from [89]]). (**D**) Illustration of the fixed scar substrate: left, example of high-definition ex vivo cardiac magnetic resonance imaging highlighting the infarct (top) used to reconstruct the infarct in 3D (bottom); right reentrant mechanism of tachycardia utilizing channel of surviving myocytes in the BZ (Adapted from [90]).

Clinical and experimental mapping and ablation studies have greatly advanced our understanding of the in vivo arrhythmogenesis in ICM. The current mechanistic framework is that underlying and pre-existing vulnerability is created through structural and functional abnormalities. In the post-MI heart, this is classically due to the scar formation and LV remodelling with creation of the unique BZ. On this substrate, initiating events, e.g., adrenergic activation, ischaemia, mechanical loading and electrolyte abnormalities, create the trigger for arrhythmias, typically a PVC (Figure 4A). Progression to VT and maintenance are mostly the result of the substrate properties.

### 4.2. Triggers and Arrhythmia Initiation

Monitoring studies have highlighted that the majority of arrhythmias in ICM are initiated by a triggering-PVC [80,81]. Such PVCs can be due to automaticity, triggered activity or micro-reentry.

Automaticity is implicated particularly in acute ischaemia where injury current flows between ischemic depolarised tissue and unaffected myocardium [82]. This could depolarise injured Purkinje fibres, which develop enhanced firing and trigger PVCs [83,84].

Triggered activity from afterdepolarisations is another important mechanism of PVCs and often the consequence of increased adrenergic drive, which increases intracellular calcium [85,86]. Clinical observations that frequent PVCs can be reproduced during an EP study by infusion of isoproterenol and by high rate pacing support the link between cellular events and PVCs [42,86]. Delayed afterdepolarisations (DAD) occur during phase 4 or resting membrane potential due to spontaneous calcium release leading to calcium extrusion via sodium-calcium exchanger. The resulting transient inward current induces membrane depolarisation. If these depolarisations are large enough to reach threshold for activation of Na^+^ channels, an action potential (AP) is triggered, resulting in a PVC. Afterdepolarisations have been documented in Purkinje fibres and myocytes in ischaemia and at different stages after MI [86,87]. Myocytes within the BZ are particularly prone to DADs under adrenergic drive [88,89], and during in vivo studies in the pig with MI, we recently could demonstrate co-localisation of DADs and PVC sites within the BZ (Figure 4B). 

Another possibility is micro-reentry, e.g., within the BZ. This has not been demonstrated experimentally in ICM, but it is postulated to occur where fibrosis intersperses myocytes with variable action potential duration (APD). This would allow conduction block and re-activation in a small area <5mm creating a PVC [91,92]. In this scenario, early afterdepolarisations (EADs) associated with AP prolongation, as well as dispersion of repolarisation, in space and time, are important contributing factors.

Finally, mechano-electrical feedback in vivo, e.g., during sudden increase of afterload can be part of the initiating event or PVC [93]. At cellular level, acute stretch produces membrane depolarisations that affect the AP and membrane repolarisation or resting phase interrupting it with afterdepolarisations that can trigger APs and PVCs via calcium-mediated modulation of ion channel and ryanodine receptor function [94,95]. Classical in vivo studies utilizing monophasic action potential (MAP) recordings in human surgery and altering loading conditions by transient aortic constriction demonstrated the effects of mechanical feedback on the AP and the generation of afterdepolarisations that triggered PVCs [96].

### 4.3. Arrhythmia Substrates for Progression and Sustenance/Maintenance

The BZ has a central role for arrhythmia sustenance. The structural obstacles are classically understood to induce conduction slowing as electrical activation needs to zig-zag through the BZ maze around the scar, thereby allowing re-entry circuits to form [97]. In addition, it is increasingly recognised that functional abnormalities facilitate and are often essential to the re-entry circuit. Myocyte electrophysiology in the BZ is considered critical to the functional unidirectional block in the re-entry circuit [98,99]. Specifically, depolarisation and repolarisation differences contribute to the conduction block and allow re-activation of refractory regions allowing a re-entry loop [98,99]. In early experiments on post-MI VT mechanisms, El-Sherif and collaborators demonstrated by in vivo mapping the importance of the functional substrate and specifically dispersion of refractoriness, as well as the underlying myocyte-driven changes in ionic currents and APD [100,101]. In our recent work, we also recorded time-dependent variation in repolarisation in the BZ that likewise would facilitate re-entry (Figure 4C). Mechano-electrical coupling and regional differences in wall stress mechanics may also impact on the functional substrate and sustenance of arrhythmias. Orini et al. demonstrated using a multielectrode myocardial sock the induction of spatial inhomogeneity of repolarisation due to alterations in loading conditions that may contribute to the arrhythmia substrate and in a case report elegantly mapped the transition to arrhythmia during ischaemia in an ICM patient [102,103]. The understanding of impact of mechano-electrical feedback on electrical and mechanical activity has also been advanced greatly by the studies of mechanical dyssynchrony. Here mechanical inhomogeneity linked to ECG parameters of abnormal repolarisation is greater in ICM patients with documented arrhythmias and improves SCD prediction [104].

Recent advanced imaging combined with computational studies in experimental MI large-animal models has allowed description of the heterogeneous fibrosis and myofibre disorganisation that is characteristic of reentry circuit areas [105,106]. This data has led to novel clinical ablation strategies based on detailed imaging analysis to predict arrhythmia ablation sites with initial reassuring success (Figure 4D) [90]. The prescribed approach to ablation of post-MI VT typically depends upon the induction of stable arrhythmia pacing manoeuvres, like pace-mapping and entrainment, to determine the ablation target [42,44]. The advances in detailed 3D electro-anatomical mapping have had a profound influence on the approach for VT ablation [107,108]. A first improvement is the possibility to tackle critical features of the substrate identified with mapping during sinus rhythm, like isthmus or channels in the border zone of the infarct, determined by heterogeneous scar and late or isolated potentials or fragmented signals [109]. Another approach is homogenisation of the substrate, independent of critical features. By ablation of the entire BZ with relatively preserved voltages on mapping (0.5–1.5 mV), the culprit region is rendered electrically inert and almost all potentials rendered <0.5 mV [44,110]. Finally, in some cases of hemodynamically unstable VT or VF storm, the triggering PVCs are located and targeted for ablation [111,112]. Of these strategies, critical substrate targeting has substantially benefited from incorporation of advanced imaging into the procedure, making future changes in standard clinical practice likely. Pre-procedural magnetic resonance imaging visualises the location and extent of the infarct and fibrosis, feeding into computational modelling to create a substrate map on which critical vulnerable sites can be identified. This information is then used to personalise the ablation strategy [96,113,114]. Novel strategies including stereotactic non-invasive ablation, rely on preprocedural imagining for procedural planning and execution [115]. Initial success and encouraging results of accurate critical isthmus prediction from MRI reconstructions promises much to be expected from these strategies.

Despite these mechanistic insights in the role of post-MI substrate and triggers gained from in vivo studies, effective and safe anti-arrhythmic pharmacological therapy is lacking. A possible hindrance to pharmacology has been neglect of the heterogeneity of remodelling in the LV. Also, the multicellular environment of the BZ affects local myocyte remodelling and in vivo electrical function, factors that also need to be taken into account when designing molecular therapy.

## 5. Cellular Remodelling in the BZ Underlying In Vivo Arrhythmogenesis

### 5.1. Animal Models for Post-MI Remodelling

When considering the time-dependent remodelling in vivo, the confounders of therapy and concomitant disease, and the limited access to human tissue, it is clear that animal models remain a cornerstone for the study of post-MI changes in cardiac myocyte properties. Myocyte remodelling after MI has been studied for decades, leading to progressive insights, but also confounding the field and potential translation because of the wide variety of models that have been used [116]. Variety has been in species, type of intervention to create MI and timing of the observations at myocyte level and the lack of large data sets that are inherent to patch clamp studies. Studies of the 1980s and 90s mostly used rats in a surgical coronary ligation model, whereas the dog was the typical large-animal model for MI, created during surgery. Rat studies allowed more time points to be investigated and ligation typically induces large infarcts with extensive remodelling, eventually leading to heart failure. Nowadays, creation of MI in rats and mice after ligation, is often used as a model for heart failure. An important limitation of these small-animal models for the study of BZ and remote regions, is the difficulty of regional sampling for functional single myocyte studies and consequently, mostly data come from an aggregate of cells isolated from surviving myocardium. Some of the early data on cell morphology, nevertheless already indicated the presence of regional differences in hypertrophy between near-MI and remote regions [117,118] as also recently seen for the loss of T-tubules in the rat model [57]. The dog model allowed more precise regional sampling but typically had a short observation window, linked on the one hand to logistics and on the other hand to the limitation of the extensive collaterals and smaller infarcts. For these and other reasons, since the 2000s, the pig is becoming a leading animal model [119]. Pig cardiac anatomy and physiology is closer to humans, and infarcts can be created through ischaemia-reperfusion without surgery [116,119]. As well, surgical, and non-surgical, interventions have been developed to study chronic coronary stenosis and hibernation [120,121]. The breeding of transgenic animals with human histocompatibility genes for xenotransplantation [122,123] further increases interest in this animal model, which can be studied in vivo using clinical tools for EP studies, imaging, etc. Limitations are the logistics of handling adult pigs during long-term experiments. Logistically, sheep are easier to handle but like the dog, have coronary circulatory differences compared to man, and typically more than one coronary branch needs to be occluded to obtain large infarcts. The rabbit has been an intermediate size animal model, in terms of advantages and limitations [124,125].

In the dog and pig, different modes of MI induction have been used. In the dog, rich collateral circulation requires more extensive interventions to reduce blood flow and typically requires surgery to occlude or reduce flow in multiple coronary branches. In the pig, collaterals are sparser and a temporary single-vessel occlusion suffices to induce infarction. Permanent occlusive or stenosis methods to induce MI including ligation, embolisation and flow-limitation, in contrast to occlusion-reperfusion techniques such as balloon-occlusion create different phenotypes of MI. The former is likely more relevant to the pre-reperfusion/PCI era where coronary lesions remained in the chronic phase. In the current era of primary revascularisation, the ischaemia-reperfusion (I/R) models are likely more representative of the clinical phenotype, though the absence of pre-existing coronary and cardiac pathology remains a major distinction. Key differences are likely to be in the infarct and BZ, which are the principle source of arrhythmias and underlying SCD in ICM. In permanent occlusion models, the lack of blood-flow to the infarct and limited flow to the BZ limits infiltration and myocyte recovery. As such, larger infarcts are more observed in permanent occlusion compared to reperfusion [126]. Post-MI time-course in small-animal models, pre-clinical large-animal models and human MI are different as well [127]. In particular small-animal models exhibit a more rapid time-course and different qualitative features of cellular infiltration and signalling involved in infarct healing than large-animal models and human MI. Recent work suggests that the fibroblasts differentiation and invasion of the scar and BZ exhibit a unique phenotype and temporal profile as well [128,129].

In the last 20 years, mice have taken centre stage as animal model, mostly due to the power of genetic manipulation [126]. MI has mostly been induced by coronary artery ligation and mice are able to survive large infarcts of up to 40% of the LV. However, this often quickly progresses to heart failure and thus the ligation model is more suitable to study HF than early post-MI remodelling without overt HF [130]. More recently, I/R methods were introduced that are more relevant to study post-MI remodelling. However, the small size of the mouse heart makes it difficult to isolate sub-regions.

Finally, in the choice of animal models to study arrhythmic mechanisms post-MI, the electrophysiology of the myocytes is also important as species differences in ion currents shape the AP morphology with implications on repolarisation and the functional substrate for arrhythmias [131]. For small-animal models, there is a large disparity with the human AP, though this is less of a limitation for the rabbit compared to rat and mouse [132]. The dog, sheep and pig have an AP morphology and rate response that is close to human, but none has the exact same make-up in ion channels [133].

### 5.2. Myocyte Electrical Remodelling in the BZ–Role in Re-Entry and Triggered Activity

The work done in the early years after implementation of single cell isolation, patch clamp and calcium homeostasis recordings, has informed widely about ionic channel remodelling after MI. The many studies in rats, dogs and rabbits, are rich in detail, and summarizing them briefly here does not do them full justice. Nevertheless, it is a useful starting point before turning to the specific knowledge on the BZ, and more detail can be found in some excellent dedicated reviews [87,134,135]. An overarching feature of post-MI myocytes isolated from surviving myocardium, is prolongation of the AP with or without instability of the resting membrane potential. Underlying changes in ion currents responsible for AP prolongation are reduction of repolarizing early transient outward and delayed K^+^ currents. In cells isolated from remote regions several weeks after MI, these changes are reminiscent and probably overlap with the HF phenotype also seen in humans [136]. Ca^2+^ current is mostly reported to be reduced, but altered Na^+^/Ca^+^ exchange current can also contribute to long APs [137]. Gap junctional loss and re-organisation is another important feature of post-MI remodelling [138].

Early evidence showing region-specific and unique remodelling of cells from the BZ came from the work of Myerburg and colleagues. Using transmembrane recordings, they demonstrated that BZ myocytes had abnormal resting membrane potential (RMP), slower phase 1 (depolarisation) and either shorter or longer APD depending on the site as well as abnormal repolarisation and refractoriness [139]. Boyden and collaborators studied the early and intermediate-to-late remodelling in the dog post-MI model and also reported regional differences. Regional AP changes had a temporal pattern: in the early or acute phase (24-48 h post-MI), AP prolongation was observed consistent with reduced inward and outward K-currents [134,140]. In the sub-acute phase (5-14 days post-MI), cells studied in this intermediate phase progressively shortened AP and decreased activation velocity (measured by the maximal activation velocity of the AP upstroke, Vmax) due to a decrease in I_Na_ current and altered kinetics; however, I_K1_ appeared to be reduced in these cells. In the chronic phase described as 2-months post-MI, the AP profiles and resting potential return to normal durations comparable to control cells [141]. The cellular AP profile of the chronic or stable infarct phase after one month are likely the most relevant to arrhythmias in ICM outside of the acute myocardial injury phase. At this stage, myocytes and Purkinje cells that survive in the infarct and border zone especially with timely reperfusion, may have a special role, as also indicated by clinical recordings [83,142]. Extensive work by Boyden and collaborators also demonstrated that remodelling in Purkinje cells in the chronic phase is on the one hand particular, e.g., with regard to calcium handling (see below), but also shares features with myocytes with reduced outward potassium currents and calcium current [134,143].

In addition to these focused BZ studies, a number of early reports indicated that remodelling post-MI is heterogeneous with increased dispersion of repolarisation [144,145,146], and more recently, transmural dispersion [147]. However, few studies have directly compared cellular remodelling in the infarct/BZ region to other regions.

Table 1 gives an overview of papers that have made a direct comparison of electrical remodelling in cells isolated from more than one region in the same heart. The table is organised from top to bottom according to the time point studied after MI induction. Except for our recent paper using an I/R model, all of the studies used a permanent stenosis/occlusion. Although different in detail, they share a commonality of a BZ specific remodelling where AP is not necessarily longer, but unstable. Ca^2+^ current and transients are reduced but spontaneous events are present. Studying a late-stage remodelling time point of 5 months after MI, Hegyi et al. [88] presented a complete inventory of major ion channel fluxes in the BZ compared to the remote myocardium. In this model, inward I_CaL_ is reduced in the BZ compared to the remote region, while NCX and late-Na^+^ remained unchanged. For outward currents, I_K1_ is reduced in the BZ while I_Ks_ and I_Kr_ remained unchanged. These ion channel changes lead to regional differences in APD, with the BZ myocytes having significantly shorter APs than the remote. 

**Figure 5:**
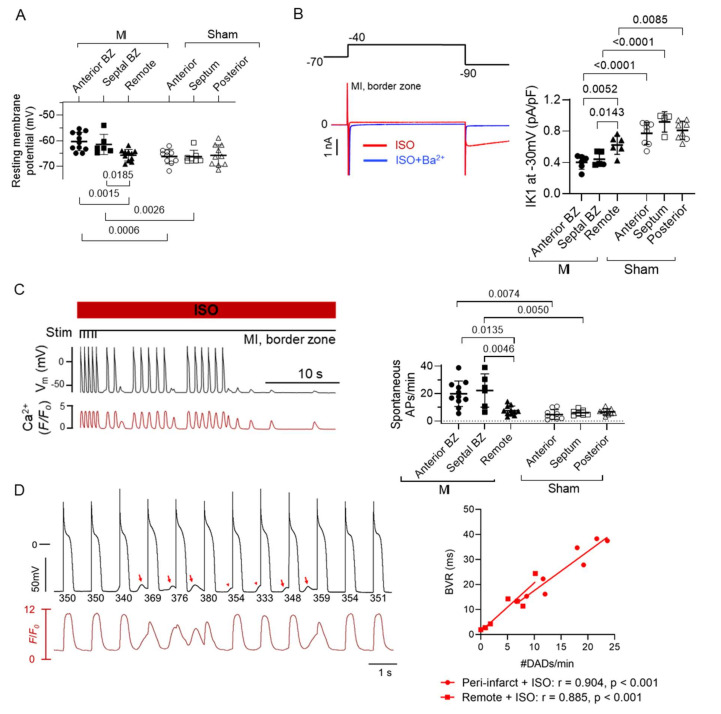
Differential regional remodelling of cardiomyocytes: propensity for DADs and triggered action potentials as well as lability of repolarisation. (**A**) Resting membrane potential (RMP) of regional isolated cardiomyocytes, border zone (BZ) cardiomyocytes have a more depolarised RMP. (**B**) Reduced I_K1_ under ISO (isoproterenol) in BZ cardiomyocytes, a contributor to depolarised RMP and propensity for triggered activity. (**C**) Spontaneous Ca^2+^ release events and triggered action potentials are increased in MI BZ cardiomyocytes during adrenergic stimulation. (**D**) Spontaneous Ca^2+^ release and delayed afterdepolarisations (DADs) influence action potential duration and resultant beat-to-beat variability of repolarisation (BVR). (Adapted from [49,89]).

Our own recent data come from an I/R model, studied at four weeks when the incidence of spontaneous arrhythmias is high [49]. Different from Hegyi et al., we did find not find shorter APs but found that the resting membrane potential was unstable with reduced I_K1_ under adrenergic stimulation, facilitating triggered APs (Figure 5A–C).

These latter findings relate to changes in calcium handling. Cells from infarcted hearts, not specifically from the BZ, were previously reported to have decreased I_CaL_ and increased ryanodine receptor (RyR)-responsiveness leading to frequent spontaneous calcium release events [137,168,169]. Also, in the pig, following a non-reperfused MI, BZ myocytes have increased spontaneous calcium release compared to remote myocytes leading to frequent DADs due to calcium extrusion via the NCX during adrenergic [89]. We recently studied the relation between cellular remodelling and in vivo events, in the I/R MI model [49]. Here we could show that myocyte-driven DAD events co-localise with the sites of origin of frequent PVCs in vivo (Figure 4B), suggesting that calcium handling abnormalities and DAD-triggered APs could be a dominant mechanism underlying PVCs originating from the BZ in the infarcted heart. Of note, DADs contribute to AP temporal variation and beat-to-beat variability of repolarisation (Figure 5D).

In Purkinje cells as well, Boyden et al. documented extensively the occurrence of calcium waves and their propagation, as a potential source of triggered activity from the BZ [170,171] corroborated by others in Purkinje fibre preparations [84]. Purkinje cells have a latent pacemaker function and calcium-driven depolarisation contributes to this automaticity, while Purkinje cells surviving post-MI have more spontaneous activity in vitro [172]. Clinically there have been recordings of premature ventricular complex (PVC) that indicated a Purkinje fibre origin. Both Purkinje cells and cardiomyocytes can have spontaneous calcium release that triggers PVCs, but whether there is a fundamental difference, or a cell preference between them as a potential PVC source, awaits a head-to-head comparison. Such abnormalities could play a central role in triggering PVCs that are central to arrhythmia initiation. Moreover, a unifying hypothesis is to consider the interplay between myocyte-DADs and Purkinje depolarisations, which could interact synergistically via parasystolic modulation to result in the manifestation of PVCs [173].

Non-myocyte remodelling contributes to the unique nature of the BZ as well. Though beyond the scope of the present review, recent studies are emerging that underscore the diverse nature of cardiac fibroblasts and fibrosis [129,174]. Fibrosis in the BZ may result from the specific myofibroblast phenotype, interacting with its environment, related to unique transcriptomic signatures as seen in pig models of MI [59,175]. Fibroblasts modulate the myocyte electrophysiology and remodelling directly through gap-junctions and through paracrine pathways, in addition to providing a collagen network that interferes with electrical conduction [135,176,177,178,179]. MicroRNA signalling from fibroblasts and other cell types is one of the modes of communication [180,181].

In summary, electrical remodelling of myocytes and the heterocellular interactions in the BZ create a unique environment for arrhythmogenesis, both as a substrate promoting re-entry and as a setting facilitating triggered events. The latter mostly result from an extra stimulus, with a prominent role for altered autonomic drive as initiating event. Hypokalaemia is another example of an initiating event, which would facilitate both trigger and substrate by inducing calcium overload as well as reduce K^+^ currents and destabilise the membrane potential.

### 5.3. Role of Autonomic Inputs in the BZ for Arrhythmogenesis

Inputs from the autonomic nervous system superimpose on the myocyte remodelling to enhance and initiate arrhythmias in several ways [64,182,183,184].

As mentioned above, innervation within the BZ is heterogeneous resulting from the denervation-re-innervation sprouting during ischaemia, reperfusion, and recovery. Early experimental studies demonstrated sympathetic innervation to be pro-arrhythmic by enhancement of the substrate by increasing dispersion of repolarisation and triggered activity that is counteracted by the anti-arrhythmic action of the parasympathetic system [146,185].

More recent studies showed that sympathetic stimulation leads to variable AP profiles in subareas of this region, reflecting the variable innervation and related modulatory actions on underlying ion channels and calcium handling [65,70,186]. Altered innervation was observed to be linked to increased transmural dispersion of repolarisation due to alterations in transient outward and inward rectifier K^+^ currents [187,188].

Cardiac myocytes have a predominance of beta-adrenergic receptors and circulating catecholamines may also contribute to or superimpose on the vulnerable substrate to promote arrhythmias. Infusion of isoproterenol has been demonstrated to increase dispersion of repolarisation in ICM patients [189]. We recently demonstrated in a pig MI model that adrenergic stimulation increased temporal dispersion, i.e., beat-to-beat variation of repolarisation, which also contributes to the functional substrate [89].

**Figure 6:**
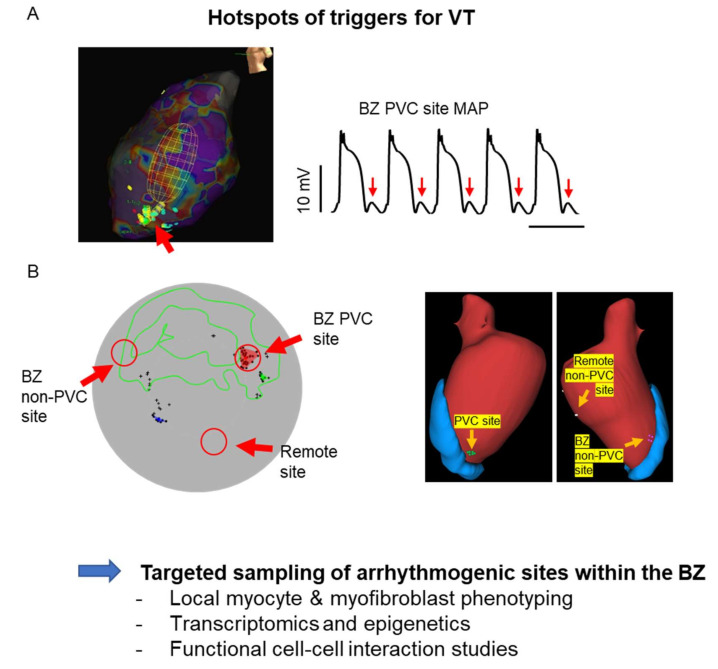
Arrhythmia site-directed studies. (**A**) Left, Electroanatomical mapping of a premature ventricular complex (PVC) preferred site, red arrow (left) and recording of the presence of triggering delayed after depolarisations at this site (right). (**B**) Corresponding polar map of the spatial localisation of arrhythmogenic and non-arrhythmogenic sites for the electroanatomical map in (**A**) (left) that can be translated to Imaging-based reconstructions to guide targeted sampling (Adapted from [49]).

Importantly, increased sympathetic tone is a major factor for initiation of arrhythmias, by generating PVCs. The link between adrenergic-enhanced spontaneous calcium release in myocytes, concurrent depolarisations through the Na/Ca exchange currents, and eventually triggered APs is well known [190]. In the BZ, the conditions to translate into a PVC are met including reduced cell-cell coupling, interspersing fibrosis, reduced repolarisation reserve and AP electrical remodelling [191]. Our recent work demonstrated this link (Figure 4), and we could identify preferred sites of PVCs (Figure 6A).

At cellular level, the adrenergic response also reflects changes in the myocyte signalling pathway. Spontaneous calcium release is mediated by Ca^2+^-calmodulin kinase (CaMK) acting on the RYRs which are differently distributed within the myocytes after MI [89,192]. Moreover, our recent data supports altered response to adrenergic signalling with increased sensitivity in the MI BZ as an underlying mechanism leading to triggered activity in the BZ [49]. Whether this relates to a reduction in receptor density or intracellular signalling is not yet established, but work from Gorelik and her group underscore the changes in receptor localisation and subtypes [193].

The autonomic modulation of arrhythmogenesis has been translated to clinical practice with the established role of first-line beta blockers in the prevention of arrhythmias in post-MI patients [16,17]. Neuromodulation is also gaining traction as studies have demonstrated its effectiveness, particularly in treating refractory ventricular tachycardia and arrhythmia storms [47,194].

In summary, the knowledge on cellular mechanisms of arrhythmogenesis in the BZ invites further translational and mechanistic research to improve specific targeting of remodelling in this region.

## 6. Emerging Concepts and Future Research Directions for Post-MI Arrhythmia Management

### 6.1. Systemic Small Molecule Therapy or Local BZ Targeted Therapy

Small molecules that prevent arrhythmias with clear benefit and few side effects are limited, and none are labelled specifically for post-MI arrhythmias. For atrial fibrillation and certain congenital arrhythmic disorders such as CPVT, research and development of small molecules modulating ion channels and transporters has been converging on Ca^2+^ handling [195,196,197]. This seems a valid direction to explore in post-MI arrhythmias as well, as far as triggers are concerned. Increased RYR activity in conditions of adrenergic stress could be mitigated through direct RYR inhibition or through the CaMK signalling pathway. Flecainide has proven its usefulness as an RYR blocker in CPVT [198] but its known proarrhythmia after MI [14], presumably related to Na^+^ channel inhibition, highlights the need for specific and safe RYR blockers. Recent work highlights the potential for modification of flecainide and removal of its Na channel inhibition [199]. Other novel RYR inhibitors have shown promise including the carvedilol derivatives [200,201] as well as tetracaine derivatives [202] and dantrolene derivatives [203] and could open new avenues for more specific targeted pharmacotherapy.

Sufficient first- and second-generation design molecules are becoming available for proof-of-concept testing in *vivo* in preclinical large-animal models. Whether targeting calcium handling also interferes with re-entry and arrhythmia sustenance is unknown. Lability of repolarisation is typically linked to K^+^ conductances but calcium-dependent changes in membrane currents can contribute as well [204,205,206]. Given that loss of K^+^ currents is common, restoring the balance between depolarisation and repolarizing currents is a challenge. Na/Ca exchange has proven a difficult target, but the Ca-dependent current mediates delayed afterdepolarisation as well as modulates the APD. Thus, when testing calcium-modulating agents, a comprehensive study of both triggers and substrate is warranted.

A major challenge for small molecule therapy is unwanted off-target effects. As well, heterogeneity of myocyte remodelling within the post-MI heart, and even within the BZ, may not align with systemic pharmacotherapy and calls for identification of suitable targets according to the location within the heart. If a unique signature of myocyte remodelling in culprit lesions could be identified, it would open new perspectives. Given the challenges for systemic pharmacology, local delivery of therapy at arrhythmic sites follows the rationale of local ablation, but without the scar formation. Despite the many hurdles, gene therapy remains an option given a suitable molecular target is identified [207]. Therefore, designing molecular therapy in line with the local mechanisms of arrhythmogenesis in the BZ requires development of more site-directed mechanistic studies (Figure 6B).

Typically, arrhythmogenic areas are identified in vivo during EP studies and recovering them for analysis ex vivo is a challenge. Imaging and computational modelling have greatly helped in identifying arrhythmic sites for ablation, but not yet in marking them for further study. On the other hand, MRI has been used to construct coordinate maps for sampling areas of abnormal contraction [208] and for local injections in the BZ [209,210]. Although these methods are not yet suitable for the recovery of arrhythmogenic sites, a combination of functional EP and MRI could in theory guide ex vivo recovery and allow studies of myocytes, as well as the tissue structure and composition.

Novel methodologies that provide deeper Insights into the local molecular mechanisms will open new perspectives. While physiological measurements at the single cell level are commonplace, only recently have we begun to understand the transcriptomic changes and their underlying regulatory mechanisms in BZ remodelling [211,212]. Transcriptomes generated from tissue is compromised by the mixture of cell types present, which through changes in cell type abundance may mask alterations in gene expression in individual cell types [211]. More recently, strategies to purify individual cardiac cell types have been developed that allow generation of cell type transcriptomes and which can provide insights into mechanisms underlying disease associated phenotypic reprogramming [213,214]. However, these analyses do not capture the diversity in phenotypes of cardiomyocytes as well as other cell types that likely arise due to the inhomogeneous cell niche environments present following MI.

### 6.2. Understanding the Complexity of the Arrhythmia Sites

Whereas myocytes are the principle actors of arrhythmias, the tissue complexity within the BZ is key to their function. Not much is known about the role of the myofibroblast/myocyte interactions in the BZ. In situ, connections between myocytes and myofibroblasts can be identified but seem to be rare [177,179]. On the other hand, ex vivo, myofibroblasts and myocytes have gap junctional connections that modulate myocyte electrical properties electrotonically. Better imaging in 3D, as currently available after tissue clearing [215], of relevant arrhythmic sites may reveal the network of neurons, myofibroblasts and their myocyte connections [61,216].

Recent advancements in single cell sequencing technology and their application to analysis of cardiac cell types have now begun to shed light on the transcriptional and cellular heterogeneity of the heart [212,217,218]. In addition to providing cell-type composition of the tissue analysed, bioinformatic interrogation of these single cell or single nucleus data sets shed light on the differentiation/disease state of the individual cell types and the trajectories taken as the various cell types transition from basal to disease state. Moreover, leveraging databases of cell ligand/receptor pairs and their assignment to the various cell types identified through sequencing, cell-cell interaction networks can be generated [217,219]. Exploitation of these identified cell-cell communication networks will yield new molecules that can enhance or prevent cell interactions. In situ transcriptomics is now providing greater context to the relative tissue locations of the cells analysed, thereby complementing more traditional tissue imaging. By in situ transcriptomics, gradients in cell population and phenotypes are identified, which may provide the information necessary to pin-point sites of initiation of arrhythmias within the tissue [212].

Single cell analysis will also contribute to identifying mechanisms by which cell phenotypes are modified in disease. In particular, analysis of the epigenome (DNA methylation, chromatin accessibility, histone modifications) in the same cell as the transcriptome uncovers mechanisms underlying transcriptional regulation including of upstream pathways [220].

Recent advances in preservation and culture of living cardiac slices [68,221,222] will allow testing in the multicellular environment and read-out of myocyte arrhythmogenic properties. As well, they can probe the contributions of cell-cell interactions. Recent data have identified coupling between macrophages and myocytes [223] calling for further studies [224] and methods development that preserve the right environment for study of interactions.

## 7. Conclusions

The management of VT/VF to prevent SCD after myocardial infarction remains challenging and calls for novel mechanistic-based non-invasive treatment.

Identification of the arrhythmia critical sites and characterisation of the molecular signature unique to these sites can open avenues to targeted therapy and reduce off-target effects that have hampered systemic pharmacotherapy. Such advances are in line with precision medicine and a patient-tailored therapy.

## Figures and Tables

**Table 1:** Regional myocyte electrical remodelling after myocardial infarction.

Study	Species	MI Stage	Disease Model	Regions	Preparation	Observations
Tsujii et al., 2003 [148]	Rat	Acute (2 h)	LAD ligation	BZ (epi) vs. remote (epi)	tissue LV (optical mapping)	Ca^2+^ waves in BZ, uniform synchronous CaT in remote.
Takahashi et al., 2004 [149]	Dog	Acute (3–4 h)	Ligation side branch of LCX (ex vivo)	BZ (epi) vs. remote (epi)	tissue LV (optical mapping)	↓ APD90, ↓ CV, ↓ APA, ↓ diastolic potential in BZ vs. remote.
Baba et al., 2005 [99]	Dog	Intermediate (5 d)	LAD ligation	central vs. outer reentry path (cBZ vs. oBZ)	single myocytes LV (whole-cell patch-clamp)	↓ I_Na_, ↓ I_CaL_, ↓ Ito in cBZ and oBZ.
Cabo et al., 2006 [150]	Dog	Intermediate (5 d)	LAD ligation	different regions of reentry path within BZ (epi): central vs. outer reentry path (cBZ vs. oBZ)	single myocytes and tissue LV (electrogram)	↓ CV longitudinal and transverse vs. normal hearts, ↓ longitudinal CV in cBZ vs. oBZ myocytes, transverse CV unchanged in cBZ vs. oBZ myocytes. ↑ Cx43 laterisation in cBZ vs. oBZ myocytes
Hund T et al., 2008 [151]	Dog	Intermediate (5 d)	LAD ligation (2h) + reperfusion In silico model	BZ (epi) vs. remote (epi)	in silico	↓ CaT amplitude, ↓ Vmax in BZ vs. remote with hyperactive CaMKII ↑ P-CaMKII in BZ vs. remote ↑ P-CaMKII at intercalated disk in BZ vs. control
Chou et al., 2007 [152]	Rabbit	Intermediate (7 d)	LCX ligation	BZ (epi) vs. remote (epi)	tissue LV (optical mapping)	↑ extrasystoles in BZ, steeper ADP restitution in BZ, ↑ pacing-induced Ca^2+^ alternans in BZ vs. remote
Mills et al., 2006 [153]	Rat	Intermediate (7 d)	LAD ligation	BZ (epi) vs. remote (epi)	tissue LV (optical mapping)	APD90 = in BZ vs. remote, ↓ CV in BZ vs. remote.
Pop et al., 2012 [154]	Pig	Chronic (4 w)	Balloon occlusion in LAD or LCX (90 min) + reperfusion	BZ (epi) vs. remote (epi)	tissue LV (optical mapping)	↓ APD90 in BZ vs. remote
Pinali et al., 2017 [155]	Pig	Chronic (4 w)	Microbead embolisation in LAD side branch	BZ vs. remote	tissue LV sampling	Cav1.2 =, BIN1 =, ↓JP2 in BZ vs. remote ↓ TT in BZ and remote vs. control, ↑ cell capacitance in BZ and remote vs. control.
Dun et al., 2004 [156]	Dog	Intermediate (14 d), Chronic (8 w)	LAD ligation	BZ (epi) vs. remote (epi)	single myocytes LV (whole-cell patch-clamp)	14d: ↓ I_CaL_ in BZ vs. remote, ↑ ISO effect in remote (presence of regional heterogeneity in adrenergic response); ↓ I_to_ in BZ vs. remote 8w: ↓ I_CaL_ in BZ and remote, no ISO effect in BZ and remote (absence of regional heterogeneity in adrenergic response); I_to_ = in BZ vs. remote cell capacitance = in BZ vs. remote
Dries and Amoni et al., 2020 [89]	Pig	Chronic (6 w)	Copper-coated stent in LAD	BZ (mid) vs. remote (mid)	single myocytes LV (whole-cell patch-clamp)	↑ DADs and spontaneous AP in BZ vs. remote, ↑ BVR in BZ vs. remote (with adrenergic signalling). Gene expression ↑ NPPA in BZ vs. remote. ↑ cell width, = cell length, = TTs in BZ vs. remote.
Kim et al., 2002 [157]	Sheep	Chronic (8 w)	LAD ligation	BZ (endo) vs. remote (endo)	single myocytes LV (whole-cell patch-clamp)	↓ I_CaL_, ↓ CaT amplitude, ↑ CaT relaxation time, ↓ contraction in BZ vs. remote. ↓ SERCA in BZ vs. remote. ↑ cell length, ↑ cell width, ↑ cell capacitance in BZ vs. remote.
Shimkunas et al., 2013 [158]	Sheep	Chronic (17 w)	LCX ligation	BZ (epi) vs. remote (epi)	tissue LV (force measurements)	↓ force development in BZ vs. remote
Wong et al. 1982 [139]	Cat	Chronic (2–7 months)	Ligation side branches of LAD and LCX	BZ (endo) vs. remote (endo)	tissue LV (microelectrode)	↓ APD90, ↓ RMP (depolarised), ↓ Vmax in BZ vs. remote
Kimura et al. 1986 [159]	Cat	Chronic (2–6 months)	Ligation side branches of LAD and LCX	BZ (endo) vs. remote (endo)	tissue LV (ion-sensitive microelectrodes)	↓ [K^+^], ↑ [Na^+^] in BZ vs. remote
Pinto et al. 1997 [160]	Cat	Chronic (>2 months)	Ligation side branches of LAD	BZ (endo) vs. remote (endo)	single myocytes LV (whole-cell patch-clamp)	↓ I_CaL_ in BZ and remote vs. control, ↓ APD in BZ, ↑ ADP in remote ↑ cell capacitance in remote vs. BZ/control
Kimura et al. 1988 [161]	Cat	Chronic (>2 months)	Ligation side branches of LAD and LCX	BZ (endo) vs. remote (endo)	tissue LV (microelectrode)	RMP =, APA =, APD90 =, APD50 = in BZ vs. remote
Weigand et al., 2016 [162]	Rat	Chronic (6 w)	LAD ligation	BZ (epi) vs. remote (epi)	whole heart (in vivo LV mapping)	↓ MAPA, ↑ heterogeneity of repolarisation, ↓ Vmax, MAPD = in BZ vs. remote
Walker et al., 2007 [163]	Rabbit	Chronic (8 w)	LCX ligation	BZ (epi) vs. remote (epi)	tissue LV (optical mapping)	↓ CV in BZ vs. remote
Dangman et al. 1982 [164]	Human	Chronic (end-stage HF)	-	BZ (endo) vs. remote (endo)	tissue LV (microelectrode)	ADP50 =, ADP100 =, Vmax =, RMP =, APA = in BZ vs. remote
Heygi et al., 2018 [88]	Pig	Chronic (5 months)	Microbead embolisation in LAD side branch	BZ vs. remote	single myocytes LV (whole-cell patch-clamp)	↓APD95 in BZ, ↑ APD95 in remote, CaT amplitude/relaxation =, I_Na_ =, ↓ I_CaL_, ↓I_K1_, I_Kr_ =, I_NCX_ =, I_Ks_ =, ↑ DAD/AP frequency in BZ vs. remote ↓ cell shortening in BZ/remote vs. control, = cell shortening in BZ vs. remote
Loennechen et al., 2002 [165]	Rat	Chronic (56 d)	LAD ligation	Remote vs. sham, Remote vs. BZ	single myocytes LV	↑ diastolic and systolic [Ca^2+^] in remote MI vs. sham; = diastolic and systolic [Ca^2+^] in remote vs. BZ ↑ cell length, ↑ cell width in remote vs. sham; = cell length, = cell width in remote vs. BZ cell shortening = in remote vs. BZ
Kilic et al., 2006 [166]	Sheep	Chronic (8 w)	LAD ligation	BZ vs. remote	whole heart (in vivo LV echo)	↓ SERCA, ↓ PLB in peri-infarct vs. remote (correlated with regional strain on echo)
Tomek et al., 2019 [167]	Rat	Chronic (8 w)	Antero-apical cryo-infarction	BZ (epi) vs. remote (epi)	tissue LV (optical mapping)	↑ alternans at longer cycle length in BZ vs. remote at baseline; ↓ alternans at longer cycle length in BZ vs. remote during adrenergic signalling

LAD—left anterior descending; LCX—left circumflex; LV—left ventricle; epi—epicardium; BZ—border zone; CaT—Ca^2+^ transient; APD—action potential duration; CV—conduction velocity; APA—action potential amplitude; Cx43—connexin-43; cBZ—central border zone; oBZ—outer border zone; P-CaMKII—phosphorylated Ca^2+^-calmodulin kinase 2; Cav—Calcium channel protein; BIN—bridging integrator; JP2—junctophilin; TT—transvers tubule; I_CaL_—long Ca^2+^current; ISO—Isoproterenol; DAD—delayed afterdepolarisation; BVR—beat-to-beat variability of repolarisation; RMP—resting membrane potential; Vmax = maximum upstroke velocity of action potential; I_to_ transient outward K^+^ current NPPA - Natriuretic peptide A; SERCA—sarco/endoplasmic reticulum Ca^2+^-ATPase; I_Na_—Na^+^ current; I_K1_—inward rectifying K^+^ current; I_Kr_—rapid-delayed rectifying K^+^ current; I_NCX_ Na^+^-K^+^ exchange current; I_Ks_—slow-delayed rectifying K^+^ current; MAPA—monophasic action potential amplitude; MAPD = monophasic action potential duration; PLB—phospholamban.

## Data Availability

Not applicable.
